# Deep Multi-Scale Features Fusion for Effective Violence Detection and Control Charts Visualization

**DOI:** 10.3390/s22239383

**Published:** 2022-12-01

**Authors:** Nadia Mumtaz, Naveed Ejaz, Suliman Aladhadh, Shabana Habib, Mi Young Lee

**Affiliations:** 1 Department of Computing and Technology, Iqra University, Islamabad Campus, Islamabad 44000, Pakistan; 2 Research Fellow, Lakehead University, Thunder Bay, ON P7B 5E1, Canada; 3 Department of Information Technology, College of Computer, Qassim University, Buraydah 52571, Saudi Arabia; 4 Department of Software, Sejong University, Seoul 05006, Republic of Korea

**Keywords:** anomaly detection, fight detection, video classification, surveillance video analysis

## Abstract

The study of automated video surveillance systems study using computer vision techniques is a hot research topic and has been deployed in many real-world CCTV environments. The main focus of the current systems is higher accuracy, while the assistance of surveillance experts in effective data analysis and instant decision making using efficient computer vision algorithms need researchers’ attentions. In this research, to the best of our knowledge, we are the first to introduce a process control technique: *control charts* for surveillance video data analysis. The control charts concept is merged with a novel deep learning-based violence detection framework. Different from the existing methods, the proposed technique considers the importance of spatial information, as well as temporal representations of the input video data, to detect human violence. The spatial information are fused with the temporal dimension of the deep learning model using a multi-scale strategy to ensure that the temporal information are properly assisted by the spatial representations at multi-levels. The proposed frameworks’ results are kept in the history-maintaining module of the control charts to validate the level of risks involved in the live input surveillance video. The detailed experimental results over the existing datasets and the real-world video data demonstrate that the proposed approach is a prominent solution towards automated surveillance with the pre- and post-analyses of violent events.

## 1. Introduction

In traditional surveillance systems [1], human experts are assigned to monitor the ongoing activities in a certain location using multi-view Closed Circuit Television (CCTV) cameras, where they report any kind of anomalies, such as fighting, to the concerned departments to avoid or to reduce the damage caused due to the violent actions or activities. On the other hand, the recent surge of CCTV has provided several opportunities to computer vision experts for useful applications, benefiting humankind and improving automated surveillance. The traditional sensing and functionalities of surveillance systems are replaced by automated techniques that are used to analyze the video data from CCTV cameras by considering different features and motion patterns to decide the nature of the events occurring in real-world environments. These are video classification [2] techniques to detect and to recognize different types of actions and events in surveillance videos [3], while broadly, the activities cover normal motion patterns such as walking, and abnormal actions such as punching, etc. In real-world surveillance environments, abnormal events carry more importance, as they are directly related to the safety of the citizens.

Violence detection (VD) [4,5], broadly falling under the umbrella of the anomaly detection and recognition domain, is a hot topic of research among computer vision experts due to its key role in various applications [6]. For instance, vision-based VD is a key item to provide several secure surveillance applications. Video surveillance is continuously being used to analyze and to detect the behaviors of humans, corresponding to the actions being performed by them. The major objective of VD is the identification of violence in a very short amount of time using automated soft computing techniques. Since then, the definition of violence is subjective; therefore, it is a very challenging domain from an applications perspective, as well as on a research level.

Mainstream VD methods are mostly concerned with motion patterns in the input videos, as violence happens in a sequence of frames; therefore, state-of-the-art techniques decide the nature of video frames as being violent or normal, based on sequential data analysis. Although some baseline research methods consider low-level features such as hough forest, using the Lagrangian function, among many others, to distinguish between the normal and violent patterns, they have limited performance due to their poor representation abilities. Recent techniques, however, completely rely on deep learning techniques, where researchers usually employ either spatial, temporal, or spatio-temporal features for VD. The spatial deep learning techniques consider frame-level features to classify a single input frame as being violent or non-violent. As defined earlier, VD occurs in a sequence of frames; hence, a decision based on a single frame is biased and thus shows a limitation of performance. Particularly, these methods are adversely affected by instantaneous changes in motion patterns, which cause blur and occlusion effects in the input frames. In contrast, the temporal methods are based on 3D convolution neural networks (CNNs), considering a sequence of frames to detect violent patterns in an input video. The number of frames in a sequence varies from method to method, but mostly, the researchers consider 16 frames in a single sequence. These methods show a better performance in many cases, as their decision is largely inspired from motion patterns analysis, which is an excellent parameter for consideration in a VD task. However, the temporal techniques ignore the importance of spatial details that are crucial for a complete visual understanding of an ongoing scene. For instance, the spatial features highlight the role of the background and the foreground in an input frame, thereby contributing to the final decision making process. Finally, the spatio-temporal models consider spatial as well as well as temporal dependencies from the input visual data, therefore improving the overall performance.

Despite the major challenges such as a difference in illumination and occlusion, among many others in surveillance videos-based VD, the aforementioned mainstream methods perform well in many scenarios. However, there are certain limitations that are associated with the existing spatio-temporal features-based VD methods. Primarily, the existing techniques, while considering the spatial features, rely only on the deeper layer features, while the intermediate representations are ignored by these methods. This is why most of these methods show poor results in challenging scenarios with occluded subjects and a partial appearance of the action performer. The main reason for this is that intermediate features contain mid-level spatial information such as details about the shapes of the objects, while the final layer features only contain global frame-level representations. The next big problem in the existing methods is only detecting the violence for a specific sequence of frames, where there are certain chances of mis-predictions by the model, but once the violence is detected, the integrated system raises an alarm, which could be a false alarm in many cases. Furthermore, the existing systems only predict violence without any history maintenance or visualization techniques to assist surveillance experts in analyzing predictions in detail for post- and pre-VD observations. In order to advance the VD domain and to ensure its ease of deployability in real-world scenarios, we present the following highlighted contributions to the research community.

- A novel spatio-temporal VD method considering multi-scale deep features from the spatial domain to tackle the partial appearance of the action performer and to handle the occluded violent actions.
- We consider the concept of control charts in the VD domain to analyze the violent behavior very well, to reduce false alarms, and to maintain a proper history of the events that occurred. To the best of our knowledge, we are the first to employ visualization using deep models-based VD.
- Extensive experimental results are performed on benchmark datasets to prove the effectiveness of the proposed VD model.

The rest of the paper is divided into four sections. Section 2 is related to the existing achievements in the VD domain, and their pros and cons are explained. The proposed method’s technical details are explained in Section 3. Experimental results are given in Section 4, and finally, the overall manuscript is concluded in Section 5.

## 2. Related Work

The occurrence of abnormal activities, including human violence, is rare; therefore, automated video surveillance significantly reduces labor and time waste [7]. Automated video surveillance using VD techniques is an interesting research domain for computer vision experts [8]. Therefore, there have been significant amounts of research in the VD domain in an effort to mitigate the crime rates using efficient identification.

Considering the type of classifier being used to distinguish violence from normal video patterns, the VD domain is broadly divided into two categories, including machine learning (ML)- and deep learning (DL)-based methods. The ML methods are abundantly applied in baseline research contributions, while in the last decade, DL models are widely employed in the VD domain.

**ML Methods for VD:** In order to detect violent actions within videos, pixel-by-pixel differences on consecutive frames are used as descriptors to describe the movements in a sequence. The work proposed in [9] introduced the motion blobs computed using the difference between the two positions. After that, the authors applied binarization and clustering techniques on those pixels, and represented them through non-zero pixels. Based on their centroids, the following steps only consider the K largest ones. An analysis of blob size between consecutive frames can be used to estimate their speed. Motion blobs can be used to distinguish fight and non-fight sequences based on the features extracted from them. The detection of vandalism can also be achieved by analyzing the movement detected in the video, irrespective of the number of people in the video. The Gaussian Model of Optical Flow (GMOF) is proposed in [10] to identify candidate regions where violent activities are likely to occur. When violent acts are observed, they should be viewed as deviations from what the crowd is normally doing. To classify violent and non-violent frames, the Histogram of Optical Flow (HOF) features descriptor is used for features extraction, and Support Vector Machines (SVM) is used for classification. An interesting use of optical flow is presented [11] for estimating the optical flow between consecutive frames in a sequence by using a descriptor called Violence Flows (ViF). This descriptor gathers the most significant information, and the SVM machine learning algorithm is used to classify a video as being violent or non-violent. Using the MediaEval dataset [12], the authors categorized videos into subclasses related to violence. During the training process, an SVM classifier is trained on this information, along with additional video, audio, and image features. This method is not limited to the training dataset, so it can be used on other videos that do not have any labels attached. This is also not related to the movement of the video, but rather, the content of the video that determines the method to be followed. The machine learning features extractors and classifiers have lowered generalization potentials; therefore, complex scenes with complicated motions patterns corresponding to the violent classes are not detected very well when using these techniques. Therefore, to overcome the features engineering issue and generalization potentials, deep learning models come into play, and are discussed in the coming section.

**DL Methods for VD:** An entire video sequence is summarized in a single grayscale image describing its movement content in the method presented in [13]. Next, a convolutional neural network with two-dimensional representation is used to classify the image. Two video detection schemes are presented in [14], based on 3D ConvNet [15], which is capable of learning the spatiotemporal characteristics of a video without prior knowledge. Three-dimensional convolutional neural networks consist of a 2D network with grayscale input frames, whereas the third dimension is the time information. The problem is solved in several ways by combining different solutions. A temporal and spatial ConvNet stream are employed in [16] to describe violent movements based on the trajectories of the movements, and an analysis of the scene with strong deep learning features is implemented. As well, the authors in [17] analyze both temporal and spatial changes and introduce an architecture called convLSTM, which combines a convolutional neural network with an LSTM (Long Short-Term Memory). Video frames are fed into the convLSTM architecture for classification as violent or not. Followed by [18], the researchers presented a deep learning-based method for detecting violence in video footage. The features are extracted from CNN architectures such as VGG16 and Xception [19], then the authors used the Fight-CNN model to identify fights based on whether frames are labeled as fights or not. They used the Bi-LSTM to classify information based on how past and future sequences of information are related. Then, an additional layer of attention determines the significant regions. The researchers of [20] proposed a framework-based lightweight CNN with residual sequential learning technique for anomalous events detection. However, their method considers directly embedded features from the backbone lightweight model, which results in an under-representation of the input frame. This practice of utilizing deep features directly without any post analysis or refining mechanism is very common in the existing literature [7,21,22], which comes with the main disadvantage of features dependency over the existing general categories images, rather than focusing the attention towards the contents of the images from an objects details perspective, which are considered in our method using multi-headed attention and a convolutional head mechanism.

## 3. The Proposed Framework

**Figure 1:**
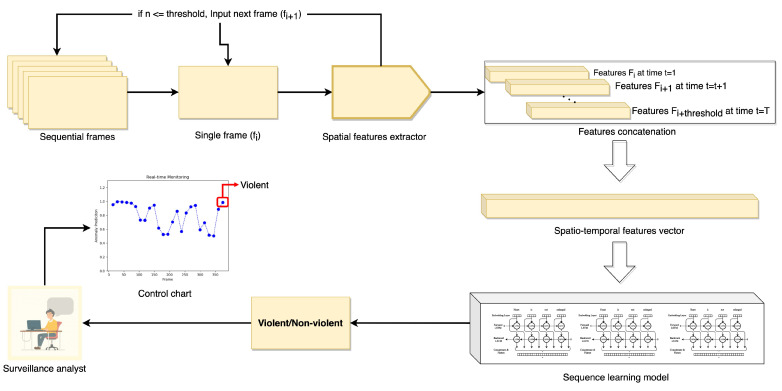
The proposed deep multi-scale features fusion attention-based network for violence detection.

The complete working procedure of the proposed deep features network accompanied by the attention mechanism and the sequence learning model is explained in this section. The proposed framework majorly comprises the newly introduced spatial features extraction model, the spatio-temporal learning sequence prediction model, and the control charts construction mechanism. These major modules are visualized in Figure 1 and are elaborated in detail in the subsequent sections.

### 3.1. Spatial Features Extraction

It is a common practice in many video classification tasks such as human activity recognition [23] to utilize an existing deep learning model’s pretrained weights in an encoder module to extract learned spatial features. These spatial features are then stacked together for a specified number of frames (defined as a single sequence), forming a unified huge sized representation of the input sequence of frames. The sequence-based feature representation is then processed using a temporal learning mechanism to advance towards the final video classification task. Traditionally, these features are extracted from the final layers of different deep learning models; for instance, many video classification approaches can be observed in a spatiotemporal learning-based review article [23]. It is evident from these articles that the features extracted from the backbone model are not processed further; rather, they are directly fed to the spatiotemporal deep learning model. This is why these models’ final classification results are not generalizable.

In this framework, we rethink the encoder-based existing model’s features and replace the traditional working of the spatial features extraction module. In the proposed encoder (ϵ), the deep features ($F_{\alpha }$) from an existing backbone model are further refined to highlight the most important contents and to select significantly contributing details from an input frame. The feature vector $F_{\alpha }$ contains textures and shapes information that are generic to the categories of the objects present in ImageNet. Since the features are extracted using the CNN mechanism, the values representing an input frame are therefore highly static without considering the relative positions of different features. Furthermore, tracking long-range dependencies via CNN features requires large receptive fields and the existing researchers lack a focus on the design of the backbone model for VD task while extracting spatial features. On top of this, large receptive fields have several other disadvantages such as compromising the computational and statistical efficiency of the network. Therefore, considering these key points, we employ the self-attention module over the spatial features extracted from a backbone CNN model. The core elements of self-attention focus on capturing *long-term* information dependencies between the sequence elements, thereby producing better results for sequence learning problems such as VD. A self-attention module is employed over the CNN features to model the interactions between the entities of the feature representation $F_{\alpha }$, where the self-attention layer aggregates the global information from the input sequence and updates each layer [24]. Herein, we employ multi-headed attention, ϑ, which comprises several self-attention blocks, and each block contains its own learnable weights.

After processing the spatial feature maps using multi-headed attention, we acquire refined features $F_{\beta }$. The refined feature vector is further processed using a series of convolution operations to make the $F_{\beta }$ distortion invariant and to obtain the perks of CNN layers, as validated by [25]. Finally, after the convolution head, we obtain a linear feature vector, $F_{\gamma }$ that is advanced to the spatio-temporal learning mechanism for final violence identification.

The mathematical formulation of the proposed encoder’s ϵ features extraction and refining is given in Equation (2).

(1) $$\begin{array}{c} F_{\alpha }=\tau \left(I_{RGB(H\times W)}\right),\\ F_{\beta }=\vartheta \left(F_{\alpha }\right),\\ F_{\gamma }=\Lambda \left(⌀\cdots \Lambda \left(\cdots ⌀\left(\pi_{3}\left(\pi_{2}\left(\pi_{1}\left(F_{\beta }\right)\right)\right)\right)\right)\right), \end{array}$$

where τ is the backbone model, π refers to the convolution layer, *⌀* donates the activation, ⋯ represents a series of layers such as linear and convolutions, and Λ is used for fully connected linear layers.

The backbone model selection [26,27] for spatial features extraction is a very critical decision for a complex computer vision problem such as violence detection. Therefore, we carefully observe the role of various spatial features extractors in different computer vision domains. The VGG family is frequently used as a backbone for the most precise object detection models such as R-CNN, Faster R-CNN, and SSD. Similarly, the effective features representation potentials of the VGG family are analyzed in various tasks; for instance, Ale et al. [28] produced various ablation results to test the features representation potentials of backbone models. The authors conclusively presented a good trade-off between the number of parameters of a backbone model and the error rates. Therefore, considering the theoretical and experimental verification, we have used the VGG-19 model as our backbone for spatial features extraction.

### 3.2. Spatio-Temporal Learning Mechanism

The spatial features are effective for many image-based classification and recognition domains, but they show limited performance in sequence-based problems such as video classification. Therefore, in the video classification domain, sequence learning strategies are applied over the spatial features to enhance their potentials for sequential problems involving spatial object details and motion patterns learning. Spatio-temporal learning in the computer vision domain is widely adopted using Recurrent Neural Networks (RNNs), which are famous for sequential information processing using spatial and temporal sequential data effectively. RNNs were initially introduced to identify the patterns between sequential data i.e., from a sequence of frames that can lead to effective events recognition. However, later on, for long sequences, RNNs forget the information about the earlier patterns and this problem is known as vanishing gradient. This challenge is handled using Long Short-Term Memory (LSTM), which is an advanced variant of RNN, having the potentials to identify and to learn long-range dependencies.

The basic structure of LSTM contains input, forget, and output gates, where the *sigmoid* activation function is used among these gates to decide whether a gate should be closed or open. The data operate from the input unit leading to the output gates. Further detailed information about the internal architecture of LSTM is out of the scope of this paper and can be deeply studied from the existing research [29]. In the proposed framework, inspired from the role of bi-directional LSTM in action recognition domain [29], we employ it in our research. The bi-directional LSTM structure processes sequential information using two stacked LSTMS, where one processes information in the forward direction and the other one observes information in the backward direction. Finally, their integrated output is computed using the hidden layer of the forward and backward LSTM layer. The classifier performance of the LSTM is validated and back-propagated after the output layer.

(2) $$VD_{prediction}=\mu_{2}\left(\mu_{1}\left(F_{\sigma }\right)\right),$$

where μ represents an LSTM layer and the final VD is predicted from the second LSTM layer.

### 3.3. Control Charts Construction

In statistical process control, the control charts that are also known as quality control chart are normally used to identify whether a process operation is normal or abnormal. The control charts monitor the ongoing processes using upper and lower control limits. Upper control limit is called UCL, while lower control limit is known as LCL. A central line in the control chart indicates normalcy, where an ongoing operation is considered as normal or *in a state of control* when the data points are between UCL and LCL.

**Figure 2:**
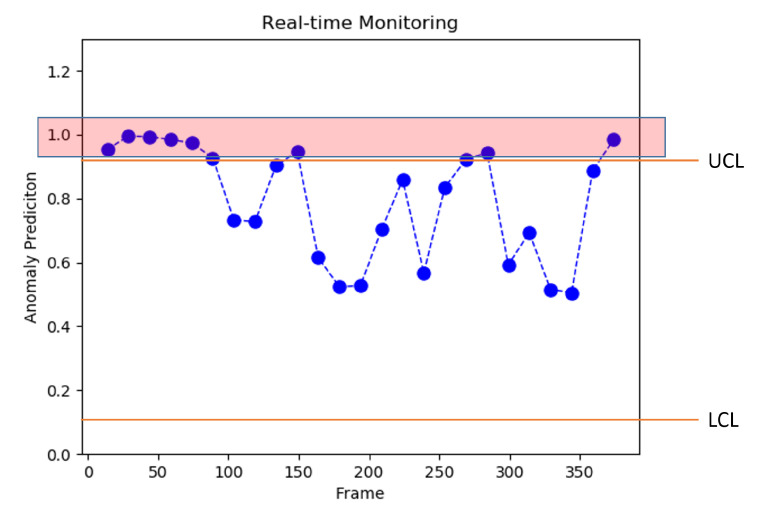
The visual representation of control charts.

In our problem, we draw a control chart for the confidence of the detected violence from the proposed deep learning model. In Figure 2, some sample frames of a video are given, and the anomaly and violence score predicted by the proposed model is given on the *x*-axis, while the time series data, i.e., the number of frames, is displayed on the *y*-axis. The area marked in red contains some frames sequences in a range from 10 to 90, where the violence score predicted by the model is greater than 0.9. The control charts are used to keep track of such records, and they also present a similar figure to the surveillance expert while the real-time surveillance is analyzed.

### 3.4. Implementation Details

Inspired from the generalized features extraction potentials of the VGG model, we have used the backbone VGG19 model’s pretrained weights. The features $F_{\alpha }$ are extracted from the input RGB image, $I_{RGB(H\times W)}$ with an output dimension of 7×7 and with 512 channels. The $F_{\alpha }$ features are then refined using ϑ multi-headed attention with four heads, which outputs the same dimension features, named as $F_{\beta }$. After the attention mechanism, the features undergo a flattening procedure through a convolution head block. This block contains three convolution layers, relu activation, two convolution layers, and a relu activation, followed by the features flatten layer, and finally, two fully connected layers with relu activation, producing 1×2048 and 1×1024 dimension features, respectively. The 1×1024 features are input to the spatio-temporal model, which firstly processes it using the initial LSTM layer $\mu_{1}$, whose output is considered as the input for $\mu_{2}$. The output of $\mu_{2}$ undergoes dropout, dense layers with relu activation, and finally, the SoftMax layer for optimal classification.

## 4. Experimental Results

The detailed experimentation results, the settings used to train and test the proposed model, and the datasets utilized for comparison against state-of-the-art (SOTA) are explained in this section.

### 4.1. Setup

**Figure 3:**
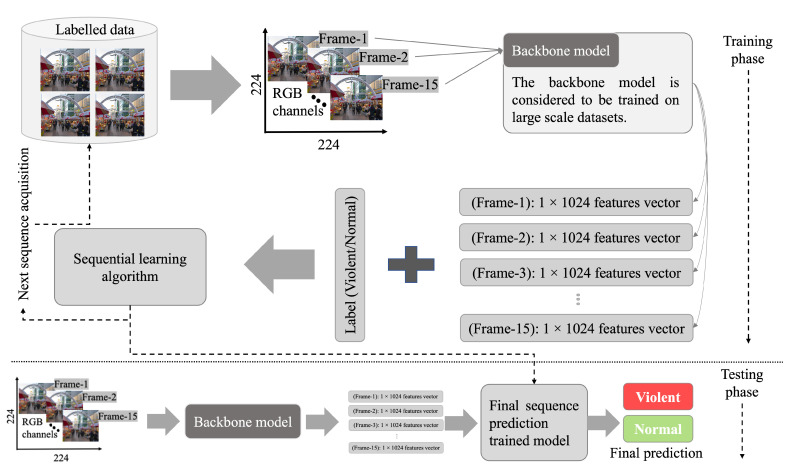
The overall training procedure of the proposed framework.

**Training details:** The proposed VD method is implemented in the famous deep learning frameworks Pytorch and Keras. The spatial features extraction is performed using Pytorch, while the spatio-temporal learning model is developed in Keras with a Tensorflow backend. It should be noted that the backbone VGG19 model weights are used to initialize the spatial features extractor, and then the backbone features are further refined using attention mechanism, followed by the convolutional head and dense layers. In the spatio-temporal learning model, *binary cross entropy* loss is used with the *Adam* optimizer. The spatio-temporal learning deep learning architecture is initialized without any backbone model pretrained weights. Furthermore, during the spatio-temporal learning model training, callbacks are implemented during training to obtain the most accurately trained model. Initially, the input frames are resized into a standard 224×224, and the initial learning rate is set as a default of 0.01. In the first LSTM layer, there are 128 units, while in the second LSTM layer, there are 64 units. We have used a Windows 64-bit operating system in an Intel(RB) Core(TM) i5 CPU and the GPU utilized for experimentation is an NVIDIA Gefore 3050 Ti laptop GPU. The overall training procedure is visualized in Figure 3. In Figure 3, the labeled data from an under process dataset are considered as input. A sequence from the training data is considered, where a single frame is first input to the backbone model to extract features. The extracted features are stacked on top of each other sequentially until the sequence reaches its thresholded limit i.e., 15 in our case. The combined features vector is attached with the relevant label to produce one hot vector, as the *violent label* features vector is shown in Figure 3. The one hot vector is input to the sequence learning deep learning model to produce a trained model that is tested in the next phase to produce *violent* or *normal* labels.

### 4.2. Datasets

The experiments are carried out using seven VD benchmark datasets from diverse categories, where some of them purely relate to real world surveillance violence and the others comprise sports and movie violence. Each of the dataset is explained in the subsequent sections. The overall details of these datasets are given in Table 1.

#### 4.2.1. Hockey Fight

Hockey fight is the most commonly used dataset [30] in the VD domain, comprising a total of 1000 short video clips that are extracted from the videos of a National Hockey League. The splitting of the dataset into fight and non-fight classes is adequately balanced, i.e., 500 videos in each category.

#### 4.2.2. RWF-2000

RWF-2000 [31] is one of the most challenging VD datasets, and it is different from most of the existing datasets in terms of the recording environment. A majority of the VD datasets are recorded in some sort of controlled environments with easily differentiable background and foreground. Furthermore, real-world violence using vision sensory data contain many challenges such as small scale objects, and different kind of resolutions and view points, etc. The RWF-2000 is generated from the YouTube repository, where the videos have only five second durations, with degraded image quality.

#### 4.2.3. Others

The other datasets that are used for experimentation include violent crowd, also known as violent flow [11], violence in movies [30], surveillance fight, and industrial surveillance [21]. The violent flows dataset is derived from YouTube videos, and the industrial surveillace datasets contains videos downloaded from YouTube, where the events include real-world environments-based violence.

### 4.3. Discussion

**Figure 4:**
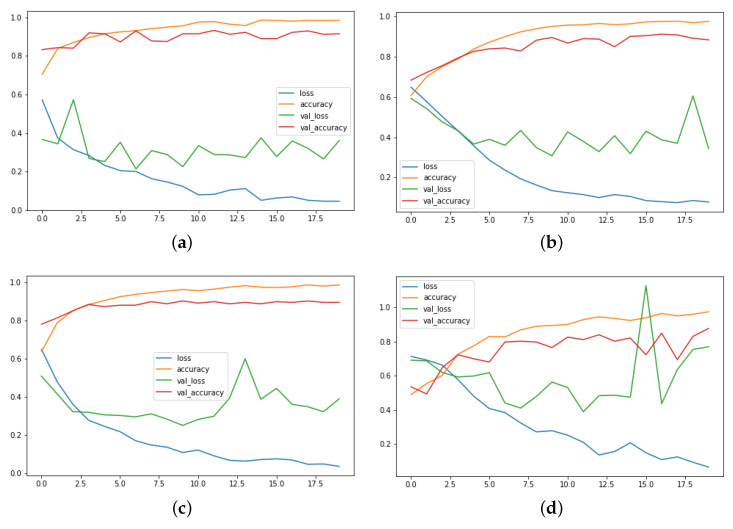
The training and validation graphs of the proposed model for different VD datasets. The *x*-axis represents the number of epochs, while the *y*-axis stands for the unit score of loss and accuracy. (Please zoom in for better reading). (**a**) Hockey fight; (**b**) RWF-2000; (**c**) Violent crowd; (**d**) Surveillance fight.

**Figure 5:**
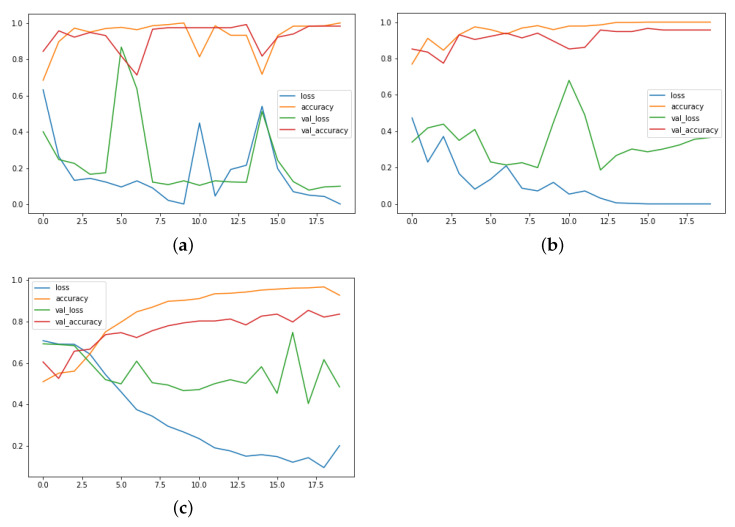
The training and validation accuracies and losses of the proposed model trained using BD-LSTM. The *x*-axis represents the number of epochs, while the *y*-axis stands for the unit score of loss and accuracy. (Please zoom in for better reading). (**a**) Peliculas; (**b**) Violence in movies; (**c**) Industrial surveillance fight.

The experimental results attained, and the training and validation graphs of the proposed method are shown in Figure 4 and Figure 5. The Hockey fight dataset training performance, as well as the validation evaluation, is given in Figure 4a. In this figure, it can be observed the training loss gradually decreases with the number of epochs, but the validation loss is somehow unstable, although converging at the final epochs. The training and validation accuracies on the same dataset increase at a constant slow pace, where it finally converges to a still value after 18 epochs. The spikes in the loss of the proposed model and the comparatively lowered accuracy for the Hockey fight dataset are due to the lack of proper long-range patterns.

The RWF-2000 training and validation loss, and accuracy graph for the proposed model is shown in Figure 4b. The training and validation accuracy line from the start of the training until the final epochs is in a stable position. In contrast, the validation loss deviates from the training in the final epochs. The RWF-2000 is a challenging dataset and the proposed model is able to model the temporal long-range dependencies in an effective manner with good accuracy.

The violent crowdviolent flow dataset performance is also stable for the training and validation accuracy, showing a stable line graph in Figure 4c. It can be verified from this figure that the proposed model provides better pattern learning abilities for the video data containing complex patters. As discussed in previous sections, the violent flow dataset resolution is very disturbed, with motion blur effects and many more challenges, but we have still achieved a significantly higher performance.

Similarly, in the other scenarios, for the surveillance fight dataset, the accuracy is comparatively lower, as well as the loss being higher due to the high-level of variability among the videos sets, as they are not recorded in some controlled environments. The graph is shown in Figure 4d.

Towards the most non-challenging Peliculas dataset, that is shown in Figure 5a, the proposed method is able to achieve a higher accuracy and a stable loss in the final epochs. Furthermore, the model’s performance gradually increases, where it converges very well until 20 epochs.

The training graph of the violence in movies dataset is visualized in Figure 5b, where we achieved stable validation accuracy and the model’s training converges gradually in the final epochs.

The industrial surveillance fight dataset’s training graph is given in Figure 5c. It is a very challenging dataset, yet the proposed model validation accuracy is satisfying enough to be implemented in real-world surveillance scenarios.

**Figure 6:**
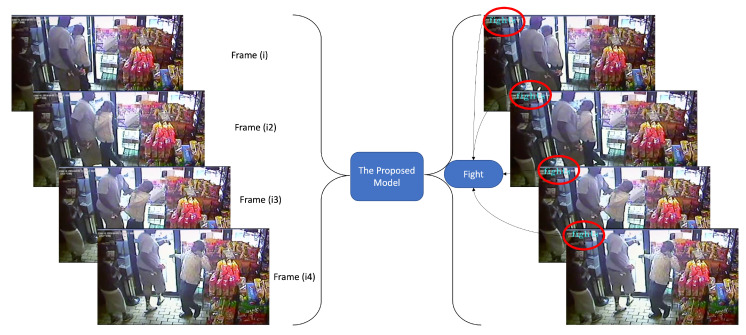
Visual output results of the proposed model over testing data from YouTube.

Some visual frames and the proposed model output results from a random fight video happening inside a shop are given in Figure 6. The first frame (i) shows the beginning of the fight scene that is captured correctly by the proposed model, although in this frame, there is a sudden transition of events from normal to violence. Therefore, this sequence of frames can be considered as the most challenging one. Similarly, the frames i2, i3, and eventually i4 also contain some sudden motion patterns with spatial information about very close objects, that makes the proposed model make a decision on the video as being fight, based on the learned parameters in the spatiotemporal deep learning model.

### 4.4. Comparison with SOTA

The comparison of the proposed model with existing SOTA techniques is discussed in this section. Overall, for most of the standard datasets, the proposed model achieved the best performance against SOTA. In some cases the proposed model’s results are lagging behind some methods, as is explained in subsequent paragraphs.

In Table 2, the performance comparison of the proposed model against the standard Hockey dataset is given. The proposed model achieved 91.29% accuracy, while for the same dataset, there are some methods achieving very higher accuracy values; for instance, Freire et al. achieved 99.4% accuracy. The Hockey fight dataset is not a very challenging dataset, and the videos given in this dataset contain very few sequences. Furthermore, there are no long-range dependencies between the frames of the Hockey fight datasets; therefore, even simple deep sequential models and spatial features are able to identify fight and non-fight classes very well. We also made experiments using a simple sequential learning bi-directional Gated Recurrent Network (GRU) to validate the phenomena that the Hocekey fight patterns are easily identifiable, and the BD-GRU performed very well, even better than our BD-LSTM model. Thus, it is concluded that the proposed model has limited performance towards the video sequences containing a smaller range of dependencies and quick actions happening in a small amount of frames. Finally, it should also be noted that the Hockey fight dataset actions are simple, where the proposed model is designed to recognize complex violent activities. The simple violent activities can be easily recognized by directly embedding the spatial features of an existing backbone model to a sequential learning mechanism. The fact is proven from the existing literature [20,21] that the direct embedding of spatial features to spatiotemporal learning has enough space for simple actions, yet limited performance for complex violent activities.

**Figure 7:**
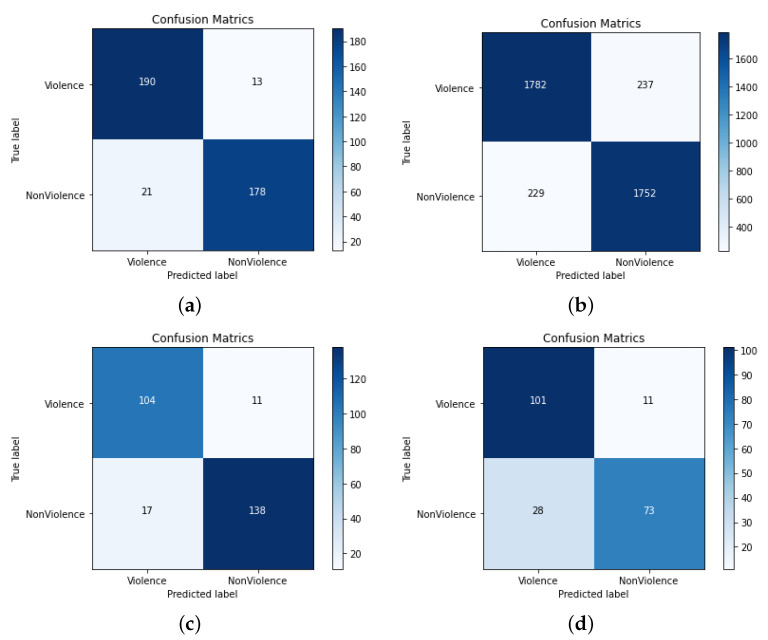
Confusion matrices of the proposed deep features attention model for VD using various benchmark datasets. (**a**) Hockey fight; (**b**) RWF-2000; (**c**) Violent crowd; (**d**) Surveillance fight.

In Table 2, we have shown the evaluation results of the proposed model against recent deep learning models. We ignore the roles of traditional models because their performances are limited for challenging datasets. The RWF-2000 is the most challenging VD dataset, comprising complex actions and sequence information among different events containing long-range temporal dependencies. It can be seen from Table 2 that the proposed model is able to identify violence patterns very well, so far achieving the best accuracy. The existing methods relying on a deep learning model’s features direct embedding have reduced performance over the RWF-2000 dataset. For instance, Ullah et al. [21] and Cheng et al. [31] have scored the second- and third-best performances over the RWF-2000. Ullah et al. [21] achieved comparatively better performances against other methods due to the role of Convolutional LSTM’s better learning abilities for sequential patterns. However, this method did not have the best performance against our model due to the absence of spatial information and features consideration. The proposed model has the ability to focus on spatial level object details, as well as the learning potentials of long-range dependencies. The confusion matrix using RWF-2000 is shown in Figure 7b, which indicates that most of the sequences are predicted correctly as violent, and similarly, the other non-violent classes. It is worth noting that the violence class is more confused with normal in the confusion matrix because some of the events happening in the RWF-2000 dataset are very challenging to identify as normal, due to their motion patterns.

Additionally, the experimental results over other datasets comprising violent crowd, industrial surveillance, surveillance fight, peliculas, and violence in movies have witnessed a stable performance by the proposed model for the VD tasks. The proposed model achieved convincing and balanced results for all of the datasets. It should be noted that instead of using multi- or dual-stream CNNs [40], we achieved a higher accuracy with a simple neural network by utilizing the backbone features effectively. The usage of spatial features following the attention mechanism in the VD domain is not very common. To the best of our knowledge, we are the first to utilize refined spatial features from a backbone model’s weights in an effective manner.

**Figure 8:**
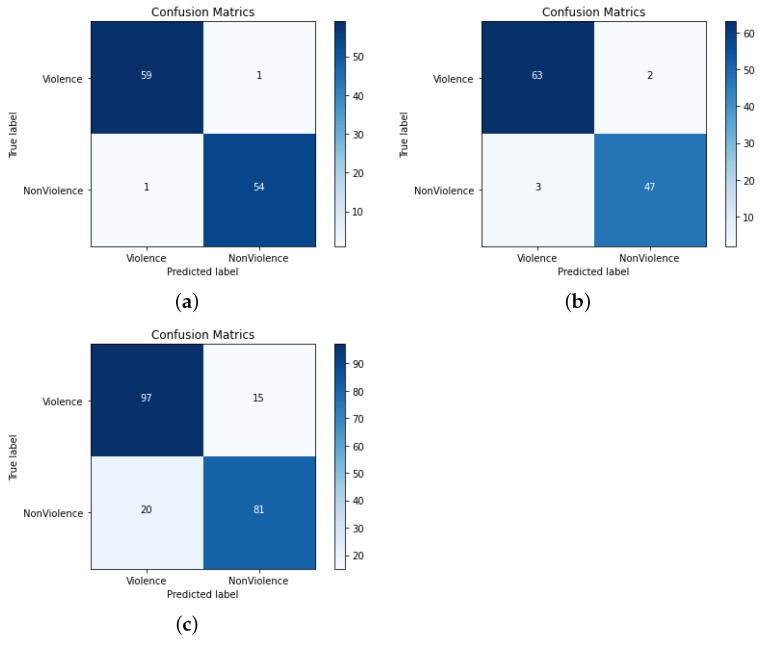
Performance analysis of the proposed model using standard VD datasets (zoom in for better visibility). (**a**) Peliculas; (**b**) Violence in movies; (**c**) Industrial surveillance fight.

The confusion matrices of the proposed model for all the datasets are given in Figure 7 and Figure 8. The performance analysis indicates that the proposed model is able to detect violence activities very effectively. In the Hockey fight dataset, the proposed method only mis-predicted 13 and 21 sequences as non-violence and violence, respectively, while in the RWF-2000 dataset, the mis-predictions are comparatively higher. On all other datasets, the proposed model is able to detect violence effectively with a reduced amount of mis-predictions and confusing outputs for the predicted classes against the actual classes.

## 5. Conclusions

Vision sensory data is utilized in the proposed research article for effective VD and its useful visualization, which assists surveillance experts. Although there are many research contributions using spatio-temporal modeling techniques for accuracy in VD, their performance is limited from a real-world feasibility perspective. Therefore, considering the challenges encountered in real-world surveillance environments such as false alarms and poor image quality, among others, we presented a novel deep features attention network and spatio-temporal learning model for effective VD. We extracted spatial features using a customized encoder for excellent frames representation potentials, followed by the widely followed BD-LSTM structure for optimal VD. The detected violence is analyzed using a famous process improvement technique called control charts, to eradicate or to reduce the false alarms in the VD domain.

With these contributions, we feel that the current research can be further improved using embedded vision technologies for a better practical implementation of the proposed system.

## Figures and Tables

**Table 1:** Statistical details of the datasets used to analyze the performance of the proposed VD model.

Dataset	# Videos	# Violent	# Non-Violent	FPS
Hockey fight	300	150	150	20–30
RWF-2000	2000	1000	1000	
Violent flow	246	123	123	25
Violence in movies	1000	500	500	25

**Table 2:** Performance evaluation and detailed comparison of the proposed model with the state-of-the-art violence detection techniques (red indicates the best accuracy and blue the second-best results, while o represents Not Available).

Method	Accuracy (%)			
	Hockey Fight	RWF-2000	Violent Crowd	Industrial Surveillance
Hassner et al. [11]	58.20	o	81.20	o
Ding et al. [14]	91.00	o	o	o
Bilinski et al. [32]	93.40	o	o	o
Mabrouk et al. [33]	88.60	o	85.83	o
Sudhakaran et al. [17]	97.10	o	o	o
Xia et al. [34]	95.90	o	o	o
Ullah et al. [35]	96.00	o	o	o
Carreira et al. (a) [36]	o	85.75	o	o
Carreira et al. (b) [36]	o	75.50	o	o
Carreira et al. (c) [36]	o	81.50	o	o
Traore et al. [37]	96.50	o	o	o
Ullah et al. [21]	98.50	88.20	o	83.57
Ullah et al. [38]	98.20	o	o	o
Freire et al. [39]	99.40	o	o	o
Cheng et al. [31]	o	87.25	o	o
Tran et al. [15]	o	82.75	o	o
**Ours**	91.29	90.47	89.63	81.22

## Data Availability

Not applicable.
